# Building an integrated cardiogeriatric heart failure pathway for very old adults: a four-year community case study from Hôpital La Porte Verte

**DOI:** 10.3389/frhs.2026.1877754

**Published:** 2026-08-26

**Authors:** Rémi Esser, Olivier Maurou, Marine Larbaneix, Alejandro Mondragon, Sophie Balesdent, Maureen Assayag, Marlène Esteban, Christine Farges, Vincenzo Palermo, Sophie Nisse Durgeat, Marc Harboun

**Affiliations:** 1 Cardiogeriatrics Department, Hôpital La Porte Verte, Versailles, France; 2 Cardiology Department, Hôpital Marie Lannelongue, Le Plessis Robinson, France; 3 Medical Affairs, NP Medical, Bordeaux, France

**Keywords:** advanced practice nursing, cardiogeriatrics, day hospital, frailty, heart failure, integrated care, older adults, remote monitoring

## Abstract

Heart failure pathways are often poorly adapted to very old adults, whose clinical trajectories are shaped by frailty, multimorbidity, cognitive impairment, functional decline, polypharmacy, caregiver dependence, and fragmented transitions between hospital, home, and long-term care settings. This Community Case Study describes the progressive construction, over nearly four years, of an integrated cardiogeriatric heart failure pathway at Hôpital La Porte Verte, Versailles, France. The pathway was developed iteratively through the phased implementation of complementary services, progressive clarification of professional roles, and repeated adaptation of coordination and escalation processes across inpatient, outpatient, home-based, and long-term care settings. Developed within a dedicated cardiogeriatrics department, the pathway connects acute inpatient care with structured post-discharge follow-up, remote monitoring, rapid-access day hospitals, advanced practice nurse-led coordination, hospital-at-home collaboration, long-term care facility outreach, pre-interventional geriatric evaluation, and palliative-oriented decision-making when appropriate. The central lesson is that integrated cardiogeriatric heart failure care should not be viewed as a single intervention, but as a responsive care architecture linking early detection, human triage, timely clinical response, reassessment, and continuity across settings. Remote monitoring is clinically meaningful only when connected to a trained response team and rapid-access care capacity; day hospitals function as reassessment platforms rather than simple alternatives to admission; and advanced practice nurses are key to operational continuity. The conceptual contribution of this report is to make visible the architecture linking these components, rather than to evaluate another isolated intervention. This single-centre experience provides a practice-based implementation framework for ageing health systems by identifying the organisational links, workforce roles, response capacities, and governance conditions required to connect otherwise isolated care components. Prospective multicentre and health economic studies are needed to assess clinical impact, scalability, cost-effectiveness, and transferability.

## 1 Introduction

Heart failure is one of the most frequent and burdensome cardiovascular conditions in older adults. Its prevalence increases markedly with age, and very old patients now represent a growing proportion of individuals living with heart failure and experiencing recurrent hospitalisations. In this article, “very old adults” refers pragmatically to the population typically managed in the department, predominantly patients aged 80 years and older, while recognising that chronological age alone does not define cardiogeriatric complexity. Cardiogeriatrics is defined here as an integrated approach combining cardiovascular expertise with geriatric assessment and decision-making, including consideration of frailty, cognition, functional status, nutrition, multimorbidity, treatment burden, caregiver capacity, and patient priorities. However, heart failure in advanced age is rarely a purely cardiac condition. It is commonly associated with frailty, multimorbidity, cognitive impairment, polypharmacy, malnutrition, functional decline, and social vulnerability, all of which influence prognosis, treatment tolerance, self-care capacity, and the ability to navigate complex healthcare systems (1–4). In this population, conventional disease-centred pathways are often insufficient because they primarily address cardiac decompensation, whereas clinical outcomes are also shaped by geriatric vulnerability, care fragmentation, and functional reserve.

Hospitalisation remains a central event in the trajectory of older adults with heart failure, but it may also become a source of harm. Acute inpatient care is sometimes unavoidable, particularly in severe congestion, renal dysfunction, hypoxaemia, or diagnostic uncertainty. Yet repeated admissions expose frail older patients to delirium, hospital-acquired complications, deconditioning, loss of autonomy, medication discrepancies, and disruption of community-based support (5, 6). The post-discharge period is also particularly unstable. Residual congestion, insufficient treatment optimisation, iron deficiency, renal dysfunction, limited therapeutic education, and delayed reassessment may contribute to early clinical deterioration and unplanned rehospitalisation (7–9). For very old adults, the issue is therefore not only how to treat acute heart failure, but how to organise a safe, rapid, and continuous response before decompensation leads to another full hospital admission.

This challenge is amplified by the limited applicability of conventional evidence to frail older populations. Major heart failure trials have transformed prognosis and established guideline-directed medical therapy as the foundation of care, but very old adults with multimorbidity, cognitive impairment, dependency, or advanced frailty remain underrepresented in many trial populations (10–12). Contemporary guidelines increasingly emphasise multidisciplinary care, continuity after discharge, patient-centred decision-making, and attention to comorbidities and frailty, but they provide limited operational guidance on how such principles should be implemented in everyday geriatric heart failure care (11–13).

Several care innovations have attempted to address these gaps. Remote monitoring may support early detection of worsening symptoms, but its effect depends on the organisation of the response that follows an alert. Without nurse-led triage, rapid clinical assessment, and access to therapeutic adaptation, telemonitoring risks becoming a passive surveillance tool rather than an integrated care intervention. Similarly, day-hospital pathways for intravenous diuretics, intravenous iron, treatment titration, or geriatric reassessment may reduce reliance on conventional admission, but their value is limited if they remain isolated services disconnected from post-discharge follow-up, community care, and long-term trajectories. In frail older adults, neither remote monitoring alone nor day-hospital care alone is sufficient. What is needed is a coordinated system able to link early detection, human interpretation, rapid-access reassessment, therapeutic response, education, and continuity across hospital, home, and institutional settings.

Cardiogeriatrics has emerged precisely at this interface between cardiovascular expertise and geriatric principles. It aims to integrate heart failure management with frailty assessment, functional evaluation, cognitive and nutritional screening, shared decision-making, therapeutic proportionality, and coordination across care settings (14, 15). This approach is particularly relevant when decisions involve treatment intensification, decongestion strategies, device or valve interventions, hospital-at-home care, long-term care facility management, or palliative-oriented goals. In this context, pre-interventional geriatric evaluation before structural or interventional cardiology procedures may help align procedural decisions with frailty, functional prognosis, treatment burden, and patient priorities. Cardiogeriatric care therefore requires more than adding a geriatric opinion to standard cardiology; it requires an integrated pathway designed around the trajectory of the older patient.

Over the past four years, the Department of Cardiogeriatrics at Hôpital La Porte Verte, Versailles, France, progressively developed such a pathway for very old adults with heart failure. Several components have already been described separately, including an advanced practice nurse-led cardiogeriatric model, a diuretic day-hospital programme, a post-discharge remote monitoring cohort, an intravenous iron day-hospital programme currently under review at BMC Geriatrics, a long-term care facility pathway, and conceptual frameworks on frailty-adaptive remote monitoring and palliative-oriented decision-making (16–21). Taken individually, these initiatives address specific gaps in heart failure care. Taken together, they form a broader integrated model connecting acute cardiogeriatric hospitalisation, structured post-discharge follow-up, remote monitoring, rapid-access day hospitals, advanced practice nurse-led coordination, hospital-at-home collaboration, nursing-home outreach, therapeutic education, pre-interventional geriatric evaluation, and proportional care planning.

The primary objective of this Community Case Study is to describe the architecture and operational principles of an integrated cardiogeriatric heart failure pathway for very old adults. The secondary objectives are to describe its progressive implementation, describe the main organisational challenges and adaptations associated with its development, and outline the conditions required for transferability to other healthcare settings.

The specific contribution of this report is to synthesise previously described or evaluated components into a coherent organisational model, with explicit attention to the connections between early detection, human triage, rapid reassessment, therapeutic response, and continuity across care settings. It does not report a new isolated cohort, does not constitute a systematic review, and does not aim to demonstrate comparative effectiveness.

This Community Case Study was developed through a structured description of routinely implemented service components, previously published or submitted programme reports, and descriptive activity indicators. No new individual-level patient analysis was conducted for this manuscript.

## 2 Context

Hôpital La Porte Verte is a specialised hospital located in Versailles, France, where a dedicated Department of Cardiogeriatrics has progressively developed an integrated care model for older adults with cardiovascular disease. The department was created to address a recurring mismatch in routine practice: very old patients with heart failure often require both cardiology-level expertise and geriatric-level interpretation, whereas conventional pathways tend to separate these dimensions. The local model combines acute geriatric medicine, cardiovascular assessment, heart failure management, day-hospital interventions, therapeutic education, remote monitoring, and coordination with community-based professionals.

The department mainly manages very old adults with complex heart failure trajectories, frequently marked by multimorbidity, chronic kidney disease, atrial fibrillation, malnutrition, cognitive impairment, functional vulnerability, polypharmacy, and recurrent decompensation. In this setting, clinical decisions cannot rely only on left ventricular ejection fraction, natriuretic peptides, or congestion status, but also require assessment of frailty, autonomy, cognition, nutritional status, caregiver support, treatment tolerance, and benefit–burden balance. This dual expertise also supports pre-interventional geriatric evaluation before structural or interventional cardiology procedures, including transcatheter valve interventions, coronary angiography, ablation, or device implantation (14, 15).

### 2.1 Implementation process and pathway evolution

The pathway matured progressively over approximately four years, with individual components introduced at different time points as part of continuous service development.

Implementation was therefore iterative rather than linear. Acute cardiogeriatric hospitalisation and structured discharge planning formed the initial clinical base. Remote monitoring was subsequently introduced to support earlier detection of deterioration. Rapid-access day-hospital services were then developed to provide timely reassessment and selected intravenous treatments. Advanced practice nurse-led coordination, hospital-at-home collaboration, long-term care facility outreach, and proportional or palliative-oriented decision-making were progressively integrated to improve continuity across settings.

The resulting model emerged from repeated adaptation of professional roles, escalation pathways, communication processes, and access to intermediate care. The pathway should therefore be understood as an evolving service configuration rather than as a fixed intervention implemented at a single time point.

Planning and implementation were led by Dr Rémi Esser, Head of the Cardiogeriatrics Department, in collaboration with Dr Olivier Maurou and the department's medical and nursing team. Decisions regarding the introduction or modification of pathway components were discussed within the department and submitted for institutional validation when required. The core medical implementation team initially comprised Dr Esser and Dr Maurou and subsequently expanded to include Dr Alejandro Mondragon and Dr Marine Larbaneix.

The main phases of pathway development are summarised in Table 1.

**Table 1:** Phased development of the integrated cardiogeriatric heart failure pathway.

Period	Component introduced or formalised	Main need addressed	Operational adaptation
From December 2022	Formal cardiogeriatric pathway structuring	Fragmented disease-centred care in very old adults	Combined cardiovascular and geriatric assessment, tailored education, and structured post-discharge referral
April 2023	Satelia® Cardio remote monitoring	Earlier detection of post-discharge deterioration	Human review of alerts and structured telephone triage
From April 2024	Hospital-at-home and long-term care integration	Fragmented community care and crisis-driven transfers	Shared escalation planning and multidisciplinary coordination
From January 2025	Rapid-access diuretic day hospital	Need for an intermediate option between outpatient care and admission	Rapid reassessment, biological monitoring, and intravenous decongestion
From January 2025	Iron, treatment titration, education, and frailty reassessment pathways	Need for structured reassessment beyond isolated procedures	Integrated treatment, education, multidimensional assessment, and escalation
From January 2025	Consolidation of the closed-loop model	Limited linkage between alerts, reassessment, and follow-up	Integration of remote monitoring, APN triage, rapid-access care, and updated care planning

The table describes the progressive development of the pathway. Because the model emerged through continuous service adaptation rather than through a single formal implementation protocol, some phases overlapped. Dates correspond to the introduction or formalisation of the principal components rather than to completely distinct implementation periods.

Several implementation challenges shaped the final organisation of the pathway. These challenges did not necessarily represent failures of individual components but showed that isolated services were insufficient unless they were connected to clinical interpretation, rapid response capacity, and cross-setting coordination.

First, conventional post-discharge follow-up did not always allow early identification of clinical deterioration in very old adults with heart failure. The initial organisation relied mainly on scheduled outpatient follow-up and contacts initiated by patients, caregivers, or community professionals, but this proved insufficient because clinical worsening could occur between planned assessments and was not always reported promptly. The pathway was therefore adapted through the introduction of Satelia® Cardio remote monitoring, combined with advanced practice nurse-led alert review and structured telephone triage.

Second, early detection of deterioration was of limited value when rapid clinical reassessment and treatment capacity were not immediately available. This could lead to delayed therapeutic adaptation or referral to conventional emergency or inpatient care and prompted the development of rapid-access day-hospital pathways for clinical reassessment, biological monitoring, intravenous diuretics, intravenous iron, treatment titration, and frailty evaluation.

### 2.2 Team composition, role definition, and training

At the time of the present report, the core cardiogeriatric team comprised four cardiogeriatric physicians, one advanced practice nurse, six registered nurses, and one coordinating nurse. The pathway also relied on regular collaboration with cardiologists, geriatricians, pharmacists, dietitians, physiotherapists, hospital-at-home teams, and community nurses.

Cardiogeriatric physicians provided integrated cardiovascular and geriatric assessment, supervised treatment adaptation, determined escalation or de-escalation strategies, and supported proportional decision-making. The advanced practice nurse conducted structured clinical assessments, therapeutic education, remote monitoring triage, preparation of day-hospital visits, follow-up planning, and coordination with community professionals. Registered nurses delivered intravenous treatments, performed clinical and biological surveillance, reinforced education, and alerted the medical team when deterioration or treatment intolerance was identified. The coordinating nurse organised appointments, information transfer, and communication across care settings.

During the four-year development period, the team expanded from two to four cardiogeriatric physicians and was strengthened by the integration of an advanced practice nurse and a coordinating nurse. Professional roles were progressively clarified as new pathway components were introduced. Staff preparation relied mainly on internal training, shared clinical protocols, and case-based discussions covering heart failure management, remote monitoring, therapeutic education, and day-hospital care. Role definition and workflow adaptation were discussed during departmental and multidisciplinary meetings.

### 2.3 Current implementation scale and operational organisation

Selected activity indicators are reported descriptively to characterise the current scale and operational scope of the pathway. As of April 2026, selected cumulative activity indicators included more than 1,200 individual patients enrolled in Satelia® Cardio, approximately 136 diuretic day-hospital visits, 215 intravenous iron day-hospital visits, and 30 titration or frailty reassessment visits. Over the broader pathway development period, the department recorded more than 3,400 heart failure-related hospitalisation episodes, with a mean patient age of 87 years. Hospital-at-home and long-term care facility collaborations involved approximately 373 patients. These figures characterise pathway maturation and should not be interpreted as comparative effectiveness outcomes.

In this model, remote monitoring was designed as a coordination mechanism rather than a stand-alone digital intervention, linking symptom alerts to nurse-led triage, clinical reassessment, therapeutic adaptation, rapid day-hospital access, or hospital-at-home activation when needed (18, 20). The day-hospital platform became the operational backbone of the pathway, combining outpatient intravenous decongestion (17), intravenous iron repletion within post-discharge reassessment, currently under review at BMC Geriatrics, treatment titration, therapeutic education, frailty reassessment, and review after alerts or instability.

The advanced practice nurse plays a central role through clinical assessment, therapeutic education, alert triage, remote monitoring follow-up, patient and caregiver support, preparation of day-hospital visits, liaison with community professionals, and escalation to cardiogeriatricians when needed (16). The pathway also extends beyond hospital walls through hospital-at-home services, community professionals, long-term care facilities, and dedicated cardiogeriatric rehabilitation beds within the post-acute care setting. Links with nursing homes were formalised through the CARE-HOME-HF pilot, integrating screening, telemonitoring, multidisciplinary review, anticipatory hospital-at-home planning, and proportionate decision-making for residents with heart failure (19).

**Figure 1:**
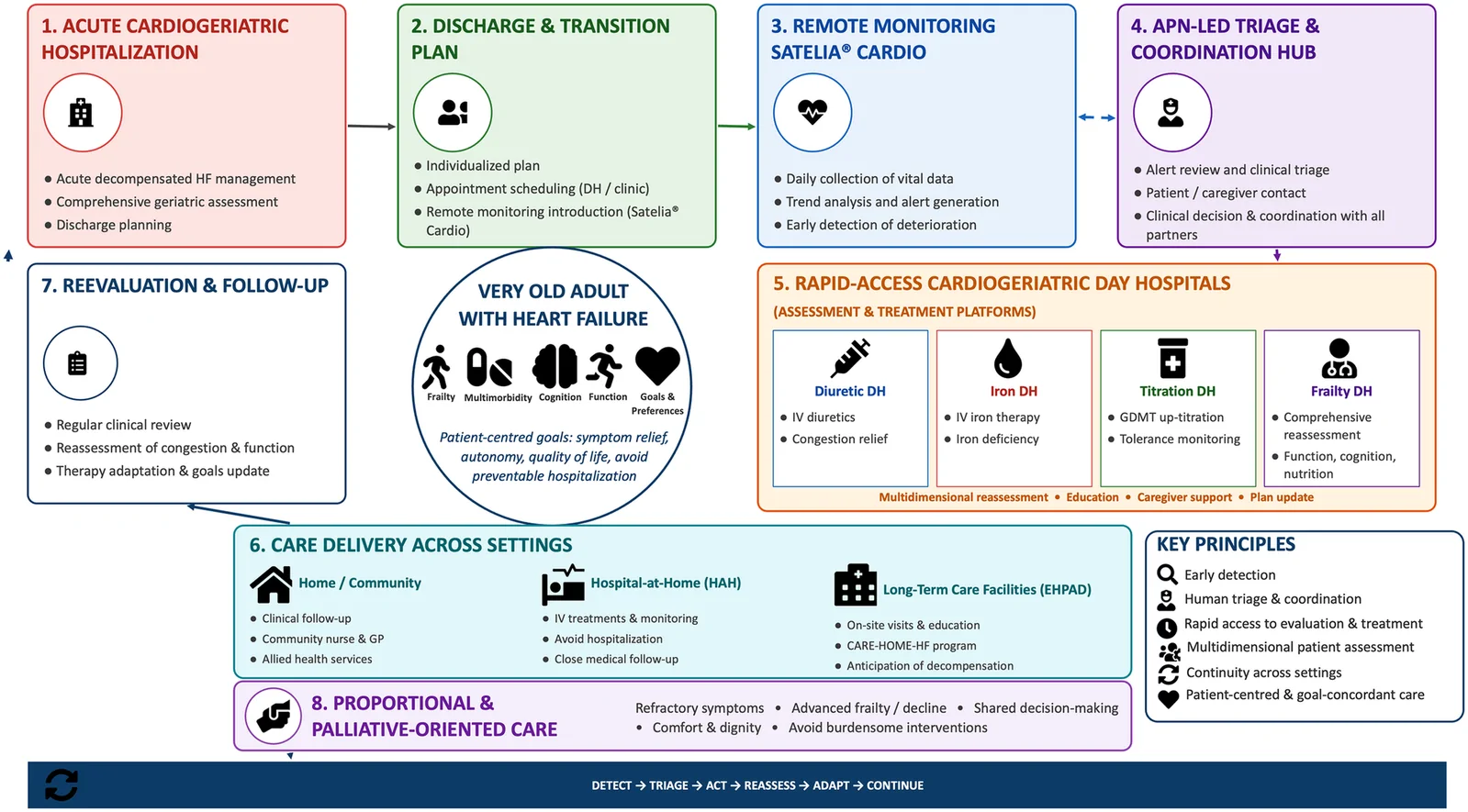
Integrated cardiogeriatric heart failure pathway at Hôpital La Porte Verte. The pathway connects acute cardiogeriatric hospitalization, discharge planning, Satelia® Cardio remote monitoring, advanced practice nurse-led triage, rapid-access cardiogeriatric day hospitals, home and community follow-up, hospital-at-home services, long-term care facilities, and proportional or palliative-oriented care. Day hospitals act as reassessment platforms for intravenous diuretics, intravenous iron, treatment titration, and frailty evaluation, within a responsive loop linking early detection, clinical triage, therapeutic adaptation, and continuity across care settings. APN, advanced practice nurse; BP, blood pressure; DH, day hospital; EHPAD, établissement d'hébergement pour personnes âgées dépendantes/French nursing home; GDMT, guideline-directed medical therapy; HAH, hospital-at-home; HF, heart failure; HR, heart rate; IV, intravenous; SpO₂, peripheral oxygen saturation.

Overall, La Porte Verte illustrates a real-world cardiogeriatric service that progressively moved from episodic heart failure management toward an integrated, frailty-informed, trajectory-based care model. The pathway connects acute hospitalisation, remote monitoring, day-hospital interventions, advanced nursing practice, hospital-at-home services, nursing-home outreach, and community follow-up into a coordinated system designed to detect instability, respond in a timely manner, and preserve patient-centred goals. The overall organisation of the integrated cardiogeriatric heart failure pathway is summarised in Figure 1.

## 3 Key programmatic elements

The main components of the integrated cardiogeriatric heart failure pathway, their target populations, clinical functions, and professional leads are summarised in Table 2.

**Table 2:** Main components of the integrated cardiogeriatric heart failure pathway.

Component	Target population	Main function	Key lead
Acute cardiogeriatric hospitalization	Very old adults with acute or worsening heart failure	Stabilization, multidimensional assessment, education, and discharge planning	Cardiogeriatrician/APN
Remote monitoring	Patients discharged after heart failure hospitalization or requiring close follow-up	Early detection, alert triage, and rapid reassessment	APN/cardiogeriatric team
Diuretic day hospital	Patients with congestion suitable for ambulatory care	Outpatient intravenous decongestion and clinical monitoring	Cardiogeriatrician/nurses
Iron day hospital	Patients with heart failure and iron deficiency	Intravenous iron repletion and post-discharge reassessment	Cardiogeriatrician/APN
Titration and education pathway	Patients requiring cautious treatment optimization	Stepwise therapy adjustment and repeated therapeutic education	APN/cardiogeriatrician
Frailty and pre-interventional assessment	Frail patients or those referred for valve interventions, CRT/device procedures, coronary angiography, ablation, or other invasive cardiac procedures	Functional, cognitive, nutritional, physiological, and benefit–burden evaluation before procedural decisions	Geriatrician/cardiogeriatrician/heart team
Post-acute, hospital-at-home, and nursing-home outreach	Homebound patients, post-acute rehabilitation patients, or long-term care residents	Home-based treatment, anticipatory planning, and avoidance of crisis-driven transfers	HAH team/APN/nursing-home physician
Proportional and palliative-oriented care	Patients with advanced frailty, recurrent decompensation, or treatment intolerance	Alignment of treatment intensity with goals, symptoms, and patient priorities	Cardiogeriatrician/APN

The table summarises the main components of the Hôpital La Porte Verte pathway, their target populations, clinical functions, and professional leads.

APN, advanced practice nurse; CRT, cardiac resynchronization therapy; HAH, hospital-at-home.

### 3.1 Acute cardiogeriatric hospitalization as the entry point

Acute cardiogeriatric hospitalization is the main entry point into the pathway. For very old adults admitted with acute or worsening heart failure, the objective is not limited to short-term haemodynamic stabilization. Hospitalization is used to confirm the heart failure phenotype, assess congestion and precipitating factors, review comorbidities, evaluate renal function and treatment tolerance, screen for iron deficiency, and perform a multidimensional geriatric assessment. Cardiac findings are interpreted together with frailty, autonomy, cognition, nutrition, fall risk, caregiver support, and expected recovery potential (14, 15).

This inpatient phase also prepares the post-discharge trajectory. Therapeutic education is initiated at the bedside and adapted to cognitive status, health literacy, sensory limitations, and caregiver involvement. Patients and caregivers receive practical information on warning signs, weight monitoring, treatment adherence, diuretic use, and escalation pathways. Discharge is therefore not considered the endpoint of hospitalization, but the activation point of a structured sequence that may include remote monitoring, rapid-access day hospital, treatment titration, intravenous iron, hospital-at-home support, long-term care coordination, or palliative-oriented follow-up.

### 3.2 Remote monitoring as a coordination tool, not a standalone technology

Remote monitoring with Satelia® Cardio has been implemented since April 2023 as a post-discharge coordination tool. Its role is not to replace clinical care, but to detect instability earlier and connect alerts to a human response workflow. In very old adults, digital alerts require clinical interpretation because worsening symptoms may reflect heart failure progression, infection, dehydration, treatment error, renal dysfunction, cognitive fluctuation, or caregiver exhaustion (18, 20).

Alerts are reviewed by the advanced practice nurse or cardiogeriatric team and generally followed by structured telephone assessment. Depending on the situation, the response may include education reinforcement, contact with the general practitioner or visiting nurse, biological testing, treatment adaptation under medical supervision, rapid day-hospital reassessment, hospital-at-home activation, or direct admission when needed. The pathway also accepts proxy-supported reporting by caregivers, home nurses, or long-term care facility staff, so that frailty or cognitive impairment does not automatically exclude patients from remote monitoring (20). In this model, telemonitoring is valuable only because it is connected to rapid clinical response capacity.

### 3.3 Rapid-access day hospitals as the operational backbone

Day-hospital programmes are the operational backbone of the pathway. They provide an intermediate level of care between conventional hospitalization and routine outpatient follow-up, allowing frail older patients to receive rapid assessment, biological monitoring, intravenous treatment, and therapeutic adaptation without systematic full admission.

The diuretic day hospital provides outpatient intravenous decongestion for selected patients with acute or worsening congestion, with monitoring of blood pressure, renal function, electrolytes, and clinical response (17). Beyond avoiding admission, each visit is used to reassess precipitating factors, chronic treatment, education, home support, and need for escalation.

The intravenous iron day hospital functions as a structured post-discharge checkpoint. Iron repletion is embedded within broader reassessment of symptoms, congestion, functional capacity, quality of life, cognition, and treatment tolerance. When congestion or instability is detected, patients can be redirected to the diuretic day hospital or reviewed by the cardiogeriatric team.

Treatment titration and frailty reassessment are also integrated into day-hospital activity. In very old adults, optimization of guideline-directed therapy requires close monitoring because hypotension, renal dysfunction, electrolyte disorders, falls, polypharmacy, and frailty may limit tolerance. Day-hospital care therefore functions not as a set of isolated procedures, but as a reassessment platform linked to hospitalization, remote monitoring, hospital-at-home, and community follow-up.

### 3.4 APN-led coordination and therapeutic education

Advanced practice nurses are central to the daily functioning of the pathway. Their role includes structured clinical assessment, therapeutic education, remote monitoring alert triage, preparation of day-hospital visits, follow-up planning, patient and caregiver support, and coordination with community professionals (16). This role requires clinical reasoning adapted to frail older adults: confirming alerts, identifying red flags, distinguishing heart failure worsening from other causes of symptoms, recognising caregiver difficulties, and escalating to the cardiogeriatrician when needed.

Therapeutic education is repeated across the pathway rather than delivered only at discharge. It covers symptoms, treatment goals, warning signs, diuretic use, adherence, diet, hydration, weight monitoring, and appropriate response to deterioration. Because many patients have cognitive impairment or functional dependency, education often targets caregivers, visiting nurses, or institutional staff as much as the patient. The advanced practice nurse also acts as a systems navigator, maintaining continuity between hospital, day hospital, home, hospital-at-home, general practitioners, private cardiologists, visiting nurses, and long-term care facilities.

Patients and caregivers were not formally involved as members of a pathway design committee or dedicated advisory group during the initial development of the programme. However, their preferences and practical constraints were routinely considered during individual care planning through therapeutic education, shared decision-making, assessment of treatment burden, and adaptation of monitoring and follow-up arrangements to cognitive status, functional dependency, caregiver availability, and patient goals. Systematic patient- and caregiver-reported experience measures were not collected during the initial implementation phase and should be incorporated into future evaluation.

### 3.5 Extension beyond hospital walls: home, hospital-at-home, and nursing homes

The pathway extends beyond the hospital because older adults with heart failure frequently move between home, hospital, rehabilitation, emergency departments, hospital-at-home services, and long-term care facilities. Hospital-at-home collaboration provides a way to deliver selected interventions outside conventional inpatient care, including intravenous diuretics, biological monitoring, nursing follow-up, hydration management, or palliative-oriented support when appropriate.

Links with long-term care facilities were formalised through the CARE-HOME-HF pilot, which integrated screening, multidisciplinary review, telemonitoring when feasible, anticipatory hospital-at-home planning, and proportionate decision-making for nursing-home residents with heart failure (19). This approach aims to support nursing-home teams, detect deterioration earlier, avoid crisis-driven emergency transfers when possible, and align escalation decisions with frailty and patient goals. Community physicians, private cardiologists, visiting nurses, pharmacists, caregivers, and institutional teams are therefore part of the same care continuum, not external actors.

### 3.6 Proportionality, frailty, and palliative-oriented decision-making

The pathway does not aim only to intensify heart failure therapy or avoid every hospitalization. In very old adults, appropriate care also requires deciding when escalation is beneficial, when it becomes burdensome, and when goals should shift toward stability, comfort, or avoidance of unwanted transfers. This benefit–burden reasoning is therefore embedded in the model (14, 15, 21).

This principle applies to drug titration, decongestion, repeated day-hospital visits, hospital transfer, device therapy, valve interventions, and palliative-oriented care. Frailty, cognition, autonomy, nutrition, caregiver capacity, symptom burden, life expectancy, and patient preferences are used to interpret whether an intervention is likely to produce meaningful benefit. Shared decision-making is applied across the pathway rather than being restricted to major procedural decisions. Treatment options are discussed in relation to expected benefit, treatment burden, feasibility at home, caregiver capacity, and the patient's priorities. When cognitive impairment limits direct participation, the team seeks to preserve the patient's previously expressed wishes and involves the designated caregiver or representative according to the clinical and legal context.

Pre-interventional geriatric evaluation before valve procedures, coronary angiography, ablation, or device implantation follows the same logic: the aim is neither to deny treatment because of age nor to intervene automatically, but to align procedural decisions with functional prognosis and patient-centred outcomes.

Palliative-oriented decision-making is considered a progressive process rather than a late binary transition. Disease-modifying and palliative approaches may coexist, especially in advanced heart failure. Recurrent decompensations, treatment intolerance, functional decline, worsening frailty, caregiver exhaustion, or repeated unwanted transfers may trigger reassessment of goals (21). In this model, proportionality and palliative integration are not separate from cardiogeriatric heart failure care; they are core components of it.

## 4 Discussion

This Community Case Study describes the progressive development of an integrated cardiogeriatric heart failure pathway for very old adults. Its main contribution is not the evaluation of a single intervention, but the description of how several complementary components were assembled into a coherent care architecture. The La Porte Verte experience suggests that heart failure care in very old adults may need to move beyond a disease-centred model focused primarily on decongestion, guideline-directed therapies, and readmission reduction, toward a care model organised around clinical trajectories, functional reserve, cognition, care transitions, caregiver capacity, and patient priorities (14, 15, 21).

### 4.1 Reframing heart failure care around patient trajectories

Conventional heart failure pathways are often structured around acute episodes, outpatient cardiology visits, and disease-specific treatment optimisation. This model is essential, but incomplete in very old adults. In this population, clinical deterioration may result from congestion, but also from renal dysfunction, malnutrition, cognitive decline, falls, medication intolerance, social isolation, or caregiver exhaustion. A trajectory-centred approach therefore requires repeated reassessment of both cardiac and non-cardiac determinants of instability.

The cardiogeriatric model presented here attempts to operationalise this shift. Acute hospitalization identifies the patient's phenotype and vulnerability profile; remote monitoring detects early instability; advanced practice nurses interpret alerts and maintain contact; rapid-access day hospitals provide clinical reassessment and therapeutic adaptation; hospital-at-home or nursing-home pathways support care outside conventional admission; and palliative-oriented decision-making helps align treatment intensity with evolving goals. The pathway therefore does not replace cardiology with geriatrics, but embeds cardiovascular care within a broader interpretation of ageing, vulnerability, and care trajectories.

### 4.2 The closed-loop model as the central organisational principle

**Figure 2:**
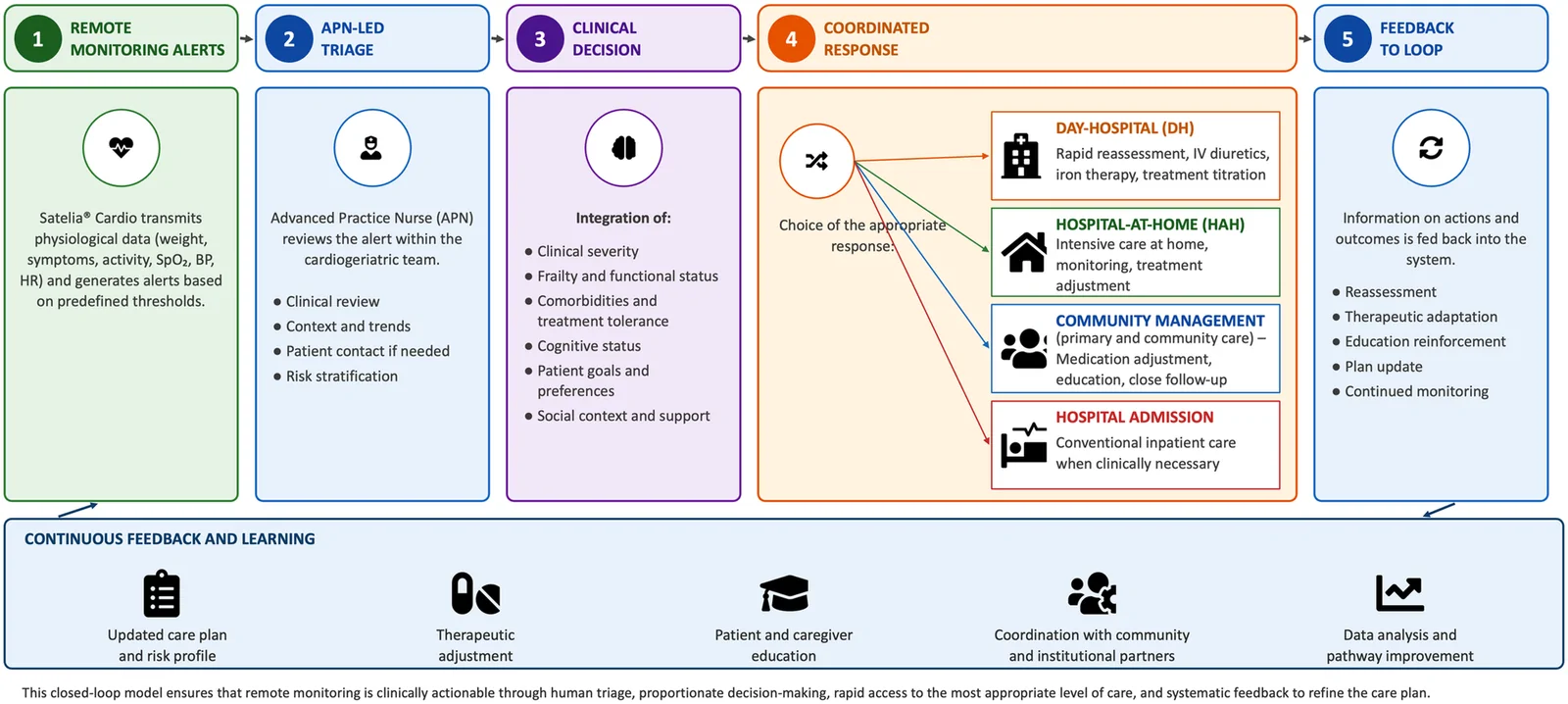
Closed-loop response model. Remote monitoring alerts generated through Satelia® Cardio are reviewed by the advanced practice nurse and cardiogeriatric team, followed by structured triage and clinical decision-making. Depending on severity, patient goals, treatment tolerance, and care setting, the response may include rapid-access day-hospital reassessment, hospital-at-home activation, community-based management, or conventional admission. Feedback is then integrated into the care plan through reassessment, therapeutic adaptation, education, and continued monitoring. APN, advanced practice nurse; BP, blood pressure; DH, day hospital; HAH, hospital-at-home; HR, heart rate; IV, intravenous; SpO₂, peripheral oxygen saturation.

In this report, a closed-loop system refers to an organisational process linking detection of clinical deterioration, human triage, timely reassessment, therapeutic response, and reintegration of the resulting decisions into the patient's care plan and ongoing monitoring. In practical terms, a remote monitoring alert may lead to advanced practice nurse assessment, day-hospital evaluation, intravenous diuretics or iron, treatment adjustment, biological monitoring, hospital-at-home activation, nursing-home support, or hospital admission when necessary. The loop is completed when the patient returns to home, long-term care, or community follow-up with updated care instructions. This closed-loop response model is illustrated in Figure 2.

This closed-loop structure is crucial because isolated interventions are unlikely to be sufficient in frail older adults. Remote monitoring without rapid response capacity risks generating alerts without meaningful action. Day-hospital care without structured upstream detection may miss the window for early intervention. Hospital-at-home services without cardiogeriatric triage may be underused or activated too late. Advanced practice nursing without access to rapid reassessment may identify problems without being able to resolve them. The value of the model lies in the linkage between components rather than in any single component alone.

### 4.3 Organisation as a therapeutic intervention

One of the main lessons of this experience is that, in complex geriatric heart failure care, organisation itself may function as a therapeutic intervention. In this model, the therapeutic value lies less in the creation of new isolated services than in the reliability of the connections between them. The “treatment” is not only intravenous diuretics, ferric carboxymaltose, treatment titration, or remote monitoring; it is the ability of the system to deliver timely reassessment, interpret clinical signals, adapt treatment safely, coordinate actors, and avoid both delayed care and disproportionate escalation.

This interpretation is consistent with the broader movement toward integrated, people-centred health services, which emphasises the need to shift from fragmented, disease-oriented delivery toward coordinated care designed around people's needs and contexts (24). It also fits with contemporary frameworks for complex interventions, where outcomes depend not only on the components of an intervention but also on implementation context, interactions between components, adaptation, and system-level mechanisms (25). The La Porte Verte pathway should therefore be understood as a complex care intervention whose active ingredients include clinical expertise, response time, multidisciplinary coordination, nursing leadership, day-hospital capacity, digital alert systems, and shared cardiogeriatric culture. Although the pathway emerged from a single institution, the relevant contribution is not the local configuration itself, but the generalisable principle that vulnerable heart failure patients require reliable connections between detection, interpretation, response capacity, reassessment, and continuity.

### 4.4 Transferability: possible, but conditional

The model is potentially transferable, but not automatically reproducible. Its implementation depends on several minimum conditions. First, the pathway requires clinicians trained in both heart failure management and geriatric reasoning, or at least a stable collaboration between cardiologists, geriatricians, and advanced practice nurses. Second, remote monitoring must be connected to a human response team; digital surveillance alone is unlikely to reproduce the same value. Third, rapid-access day-hospital capacity is essential, because early detection has limited impact if no intermediate care option exists between routine outpatient advice and emergency admission. Fourth, coordination with hospital-at-home services, community nurses, general practitioners, private cardiologists, caregivers, and long-term care facilities must be operational rather than theoretical. Finally, governance is needed to define responsibilities, escalation rules, communication channels, data monitoring, and quality improvement.

These conditions explain why the pathway cannot be reduced to a device, a day hospital, or an advanced practice nursing role. The transferable element is the architecture: a coordinated system linking detection, triage, reassessment, response, and continuity. The exact configuration may differ across countries depending on workforce, reimbursement, hospital-at-home availability, digital infrastructure, and the organisation of primary care. The main implementation lessons, barriers, operational responses, and transferability conditions are summarised in Table 3.

**Table 3:** Implementation lessons and transferability conditions.

Lesson learned	Main barrier	Practical response	Transferability condition
Remote monitoring requires human response	Alerts without timely action	APN-led triage and escalation	Trained team available for alert review
Day hospitals are reassessment platforms	Procedures may remain isolated	Clinical review, treatment adaptation, and education at each visit	Rapid-access day-hospital capacity
APNs ensure continuity	Cross-setting coordination is complex	APN-led follow-up, education, and liaison	Recognised APN role within the pathway
Frailty changes care priorities	Disease-centred care may miss patient goals	Frailty, cognition, autonomy, and goals integrated into decisions	Geriatric assessment skills
Care must extend beyond hospital	Emergency transfers may be crisis-driven	Links with hospital-at-home, nursing homes, and community teams	Operational community partnerships
Interventional decisions need geriatric input	Procedural criteria may dominate	Pre-interventional benefit–burden assessment	Heart team including geriatric expertise
Sustainability requires governance	Informal coordination is fragile	Shared protocols and regular multidisciplinary review	Defined roles, workflows, and indicators

This table summarises key implementation lessons, identified barriers, practical responses, and conditions for adaptation in other healthcare settings.

APN, advanced practice nurse.

### 4.5 Governance, quality monitoring, and sustainability

The sustainability of an integrated pathway depends not only on clinical expertise but also on clear governance. Responsibilities must be defined for alert review, treatment adaptation, day-hospital referral, escalation to hospital-at-home or conventional admission, communication with community professionals, and reassessment after intervention. Governance is particularly important because the pathway crosses conventional organisational boundaries and relies on timely coordination between multiple professionals and care settings.

Quality monitoring should combine activity indicators, process measures, clinical outcomes, and patient-centred outcomes. Relevant indicators may include the number and type of remote monitoring alerts, response times, day-hospital access, escalation to hospital-at-home or conventional admission, treatment-related adverse events, rehospitalisation, emergency department use, functional trajectories, and patient or caregiver experience.

At Hôpital La Porte Verte, pathway governance is led within the Cardiogeriatrics Department by the Head of Department and the core medical and nursing team. Operational issues are discussed during daily departmental meetings and, when required, during multidisciplinary meetings involving the relevant professionals. Routine monitoring currently focuses mainly on activity indicators, including the number of heart failure-related hospitalisations, patients enrolled in remote monitoring, day-hospital visits, and patients managed in collaboration with hospital-at-home and long-term care facilities. Continuity during staff absence or turnover relies on shared clinical protocols, multidisciplinary communication, and redistribution of responsibilities within the team.

### 4.6 Relationship with digital health implementation literature

The pathway is consistent with recent digital health literature showing that the clinical value of technological interventions depends not only on the technology itself, but also on their integration into clinical workflows, workforce preparation, patient engagement, and the organisation of care delivery (26, 27). Evidence from telemedicine applications in cardiovascular disease and broader reviews of digital health interventions also highlights the importance of linking remote technologies to professional oversight, timely clinical response, and continuity across care settings (26, 27). In the present model, remote monitoring is therefore not treated as an autonomous intervention, but as an entry point into a broader response system involving human triage, clinical reassessment, therapeutic adaptation, and feedback into ongoing care. More broadly, policy-oriented analyses of emerging health technologies emphasise the need for appropriate governance, staff training, evaluation frameworks, and implementation strategies when integrating new digital tools into routine practice (28, 29).

### 4.7 Relationship with previously published components

This article differs from the previously published and submitted reports generated by the La Porte Verte programme. Earlier reports described specific components: advanced practice nurse-led cardiogeriatric pathways, the diuretic day-hospital model, post-discharge remote monitoring outcomes, the iron day-hospital programme currently under review at BMC Geriatrics, long-term care facility integration, frailty-adaptive telemonitoring, palliative-oriented cardiogeriatric decision-making, and pre-interventional cardiogeriatric frameworks for device and structural heart interventions (16–23). The present Community Case Study does not aim to duplicate these findings or to reanalyse their cohorts. Its purpose is to describe the overall architecture that progressively connected these components into a single care pathway. The contribution of this report is not to promote a local model as evidence of effectiveness, but to use a mature single-centre experience to describe the organisational conditions required to connect remote monitoring, day-hospital care, advanced nursing coordination, geriatric assessment, and proportional decision-making into a responsive heart failure pathway. The main published or reported components informing the pathway, including several reports from the La Porte Verte programme, are summarised in Table 4.

**Table 4:** Published and reported components informing the integrated pathway.

Published or reported component	Main focus	Study/article type	Contribution to the integrated pathway
APN-led cardiogeriatric pathway	Advanced practice nursing in heart failure care coordination, education, titration, day-hospital activity, and remote monitoring	Practice-based descriptive report	Defines the APN as a central operational link between inpatient care, remote monitoring, day hospitals, patients, caregivers, and community professionals (16)
DOME-HF	Diuretic day-hospital care for very old adults with acute or worsening heart failure	Retrospective observational study	Supports outpatient intravenous decongestion as a rapid-access alternative to conventional hospitalization in selected older patients (17)
REACT-HF	Rehospitalisation patterns after heart failure hospitalization in very old adults managed with remote monitoring and structured follow-up	Retrospective cohort study	Provides real-world data on post-discharge follow-up, remote monitoring-supported coordination, and markers associated with rehospitalisation (18)
Iron-DH programme	Intravenous iron repletion integrated into post-discharge cardiogeriatric reassessment	Submitted practice-based care model, under review at *BMC Geriatrics*	Positions iron repletion as an opportunity for reassessment of symptoms, congestion, functional status, cognition, and treatment tolerance
CARE-HOME-HF	Integrated heart failure pathway for long-term care facility residents	Prospective pilot implementation study	Extends the pathway beyond hospital walls through nursing-home screening, telemonitoring, hospital-at-home planning, and proportionate care decisions (19)
Frailty-adaptive remote monitoring framework	Adaptation of remote monitoring according to frailty profiles	Conceptual framework/accepted report	Reframes telemonitoring as a stratified care strategy adapted to robust, prefrail, frail, and palliative trajectories (20)
Palliative-oriented cardiogeriatric framework	Integration of palliative principles into decision-making for frail older adults with advanced heart failure	Narrative review/conceptual framework	Supports therapeutic proportionality, goal reassessment, and palliative-oriented care as core elements of cardiogeriatric heart failure management (21)
Pre-interventional cardiogeriatric frameworks for CRT and T-TEER	Integration of frailty, cognition, functional status, physiological reversibility, and patient-centred goals into device and structural cardiology decisions	Narrative reviews/cardiogeriatric decision frameworks	Extends the pathway to procedural decision-making by supporting proportionate selection and post-procedural continuity for very old adults referred for device or valve interventions (22, 23)

This table summarises the main published or reported components informing the integrated pathway and their contribution to the cardiogeriatric heart failure model.

APN, advanced practice nurse; CRT, cardiac resynchronization therapy; DOME-HF, diuretic outpatient management for the elderly with heart failure; Iron-DH, iron day hospital; REACT-HF, rehospitalisation patterns in very old adults with heart failure managed within an integrated cardiogeriatric post-discharge care pathway; T-TEER, transcatheter tricuspid edge-to-edge repair.

This distinction is important because the present report does not reanalyse or pool the previously reported cohorts. Its originality lies in describing how individually reported components were connected into a single organisational architecture. The pathway should therefore be interpreted as an implementation model that generates testable hypotheses about coordination, responsiveness, and continuity rather than as evidence of comparative effectiveness.

### 4.8 Implications for future evaluation

The next step is not only to describe the pathway but to evaluate it prospectively. Future studies should assess clinical outcomes, patient-reported outcomes, functional trajectories, hospital use, emergency department transfers, caregiver experience, professional acceptability, costs, and sustainability. Evaluation frameworks such as RE-AIM may help assess reach, effectiveness, adoption, implementation, and maintenance across settings (30). A multicentre design would be particularly important to determine whether the model remains feasible outside a specialised cardiogeriatric department.

Until such data are available, the La Porte Verte pathway should be considered a mature practice-based model and a hypothesis-generating implementation framework rather than proof of comparative effectiveness. Its value lies in showing that care for very old adults with heart failure can be reorganised around trajectories, frailty, rapid response, and continuity, rather than around isolated disease episodes.

Future evaluation should therefore not focus exclusively on mortality or rehospitalisation, but also on functional trajectories, days alive and out of hospital, treatment burden, caregiver experience, patient-reported outcomes, and the ability of the pathway to deliver timely responses across care settings.

Future developments of the pathway are expected to further refine rapid-access reassessment for complex older patients with heart failure. Planned extensions include a cardio-nephro-geriatric day-hospital pathway for patients with combined heart failure, renal dysfunction, diuretic resistance, or complex treatment tolerance issues, and a dedicated heart failure–frailty day-hospital pathway focused on functional vulnerability, cognition, nutrition, falls, treatment burden, and patient-centred goals. These developments remain to be formally implemented and evaluated, but they illustrate the modular nature of the model and the need to adapt heart failure care to the dominant vulnerability profile of each patient.

## 5 Conceptual and methodological constraints

Several conceptual and methodological constraints must be acknowledged. First, this Community Case Study reflects the experience of a single cardiogeriatric department in one French hospital. The pathway was developed within a specific institutional context, with local expertise in heart failure, geriatrics, day-hospital care, advanced practice nursing, remote monitoring, and hospital-at-home collaboration. Its feasibility may therefore depend on resources, professional roles, reimbursement mechanisms, digital infrastructure, and care networks that may not be available in all healthcare systems.

Second, this article does not provide a global comparative evaluation of the entire pathway. Several components have been described or evaluated separately, including diuretic day-hospital care, remote monitoring-supported follow-up, advanced practice nurse-led coordination, long-term care facility integration, intravenous iron day-hospital care, frailty-adaptive telemonitoring, palliative-oriented decision-making, and pre-interventional cardiogeriatric frameworks (16–23). However, the pathway as a whole has not been tested against usual care in a prospective comparative design, and no causal conclusion can be drawn regarding mortality, rehospitalisation, emergency department use, functional decline, quality of life, caregiver burden, or costs.

Third, the supporting data come from programmes implemented at different time points, with different objectives, populations, inclusion criteria, follow-up durations, and outcome definitions. They should therefore not be interpreted as a single unified cohort or pooled without methodological caution. Selection bias is also likely, as patients referred to day-hospital care, remote monitoring, hospital-at-home services, or long-term care facility pathways may differ from those managed through conventional care.

Fourth, patients and caregivers were not formally involved as research partners in the initial design of the pathway, and patient- and caregiver-reported experience measures were not collected systematically across all components. Although individual preferences and caregiver circumstances were incorporated into clinical decisions, future development should include structured patient and public involvement and prospective assessment of patient and caregiver experience.

Fifth, the implementation process was pragmatic and was not initially guided by a prespecified implementation science framework. Consequently, some decisions, adaptations, and intermediate configurations were not documented prospectively. The chronology and lessons reported here are therefore based on the reconstruction of service development from programme records, published reports, and the experience of the professionals involved.

Sixth, transferability depends heavily on implementation fidelity and local professional culture. Remote monitoring requires trained alert review and rapid response capacity; day-hospital care requires medical supervision, nursing expertise, available slots, and escalation rules; advanced practice nurse-led coordination requires recognised roles and institutional support; and hospital-at-home or nursing-home collaboration depends on shared communication channels and mutual trust.

Future evaluation should combine clinical, organisational, economic, and patient-centred outcomes, including rehospitalisation, emergency department transfers, mortality, functional trajectories, treatment tolerance, quality of life, patient and caregiver experience, professional acceptability, implementation costs, and cost-effectiveness. Established methodological standards for economic evaluation and implementation research may help structure this next step (31, 32).

These limitations do not invalidate the model, but they define its current level of evidence. The La Porte Verte pathway should be interpreted as a mature, practice-based, hypothesis-generating implementation framework rather than as proof of comparative effectiveness. Its main contribution is to make visible the architecture, assumptions, and operational conditions of an integrated cardiogeriatric heart failure pathway that can now be tested prospectively.

## 6 Conclusion

This Community Case Study describes the progressive construction of an integrated cardiogeriatric heart failure pathway for very old adults at Hôpital La Porte Verte. Its main lesson is that remote monitoring, day-hospital interventions, advanced practice nursing, hospital-at-home collaboration, nursing-home outreach, pre-interventional geriatric evaluation, and palliative-oriented decision-making only reach their full value when connected within a responsive care architecture.

This pathway illustrates a shift from disease-centred cardiology toward trajectory-centred cardiogeriatrics, where the aim is not only to optimise heart failure treatment or reduce rehospitalisation, but also to preserve function, anticipate instability, support caregivers, adapt treatment intensity, and align care with patient priorities. The model remains descriptive and hypothesis-generating; prospective multicentre, implementation, and health economic studies are now needed to assess its clinical impact, scalability, cost-effectiveness, and transferability.

## Data Availability

No new dataset was generated or analysed for this Community Case Study. Data supporting previously published components are available in the corresponding publications or from the corresponding author upon reasonable request, where applicable.
